# The Origin of Salivary Calculus

**Published:** 1896-03

**Authors:** Henry M. Burchard

**Affiliations:** Philadelphia, Pa.


ARTICLE II.

### THE ORIGIN OF SALIVARY CALCULUS.

BY HENRY M. BURCHARD, D. D. S., M. D., PHILADELPHIA, PA.

As will be seen from the accepted name of these deposits, their main origin is recognized to be from the saliva. Every writer upon the subject of dental or oral pathology has advanced some hypothesis in explanation of their presence and manner of deposit; but, in searching for the exact *modus operandi* of their formation, it is found that none of them sufficiently explains the composition of the concretions, their color, or the occasionally odd situations in which they are found.

This much is accepted beyond cavil,—that they are in large part composed of salts of calcium (almost enterely the phosphate, and a small amount of carbonate), and combined with organic matter; the lime-salts having been held in suspension or solution in the saliva, and, for some insufficiently-explained cause, precipitated in favorable situations, and under not clearly explained conditions.

One hypothesis regarding the cause of the precipitation of the calcium carbonate has been, or is, that calcium carbonate is held in solution by virtue of the presence of dissolved carbonic acid, as a bicarbonate in the saliva con-contained in the acini and ducts of the salivary glands; and when the saliva is poured from the ducts into the oral cavity, the carbon dioxid is disengaged and escapes, and the calcium salt, held in solution thereby, is precipitated in consequence.

Another view is, that the calcium salts are merely held in suspension in the saliva, and are precipitated when the saliva is at rest in the oral cavity. This is supported by test-tube experiments.

An opinion held by the writer for some time, but never published, was as follows: it is known that the saliva contained in the salivary glands and ducts is a fluid of alkaline reaction, holding in solution salts of calcium.

It is also recognized that in most mouths the reaction of the fluid contents is acid; the alkaline secretion of the glands mixing with the acid fluid contained in the mouth, causes the precipitation of substances soluble in an alkaline and insoluble in an acid fluid. The weakness of this assumption, as a full explanation, consists in the fact that calcium phosphate is soluble in dilute acids and is precipitated by alkalies, a reversal of the conditions necessary to prove the first assumption. But still, as we see, the lime-salts of the saliva are precipitated in a dilute acid solution.

Another hypothesis, not sufficiently explanatory, is that the calcium phosphate, in the fluid at rest, is deposited and retained in pockets, or on irregularities which may be present, and form a nidus. Accepting this hypothesis, we are still confronted with the question of mode of combination of the organic matter, which forms a constant and large part of the concretions.

These several difficulties and objections have prompted the writer to some experimentation upon the matter in question, in the hope of arriving at an explanation of the several factors involved, chemical, physiological, and pathological. It is believed that; in substance, the deductions drawn are warranted by the facts presented.

There is involved, first, the fluid which furnishes the material from which the concretions are derived.

Second, the cavity into which this fluid is poured; its configuration, boundaries; the anatomical and physiological peculiarities of its component parts, and pathological manifestations in and about these parts. These latter are present, as we know, in the vast majority of cases; or at least in so great a ratio, that it is difficult to draw a line between physiological and pathological states.

Third, the composition, structure, and appearance of the substances resulting through the interaction of the preceding factors.

First, with respect to the saliva itself. It is the combined secretion of three pairs of glands and, in part, that of innumerable small glands. According to Landois, the secretion of the partoid gland contains no mucin, has calcium in solution in the form of bicarbonate, and has present the greatest amount of $CO_2$ of any of the secretions of the several glands, and, according to Foster, contains probably globulin. Upon standing, calcium carbonate is deposited, this owing to the escape of the carbon dioxid which has held it in solution as bicarbonate.

The secretion of the submaxillary gland contains much mucin, and also deposits $CaCO_3$ upon standing.

That of the sublingual gland contains more mucin than the former, and is quite strongly alkaline.

Jacubowitsch gives us the result of analysis of the mixed secretion:

| | |
|---|---|
| Water . . . . | 99.51 |
| Solids . . . . | .49 |

and of the solids:

| | |
|---|---|
| Soluble organic matter . . | 0.13 |
| Epithelium and mucin . . | 0.16 |
| Inorganic salts . . . | 0.102 |
| Potassic sulfocyanid . . | 0.006 |
| Potassic and sodic chlorids . | 0.084 |

Hammerbacher gives as the ratio of the solids in 1000 parts of saliva:

| | |
|---|---|
| Epithelium and mucin . . | 2.2 |
| Ptyalin and albumin . . | 1.4 |
| Salts . . . . | 2.2 |
| Potassium sulfocyanid . . . | 0.04 |

Kulz found that of the 7 c.c. of gases contained in 100 c. c. of saliva there was 3.5 c. c. of free $CO_2$ which could be pumped out of it.

From the list of solids it will be seen that mucin and inorganic salts form the greater amount. Of the salts of calcium different analysis show the carbonate and phosphate to be present in variable amounts, the phosphate usually stated to be in greater amount.

The next consideration is the cavity into which this combination of secretions is poured.

This is an irregularly ellipsoid cavity, open at its posterior, and divided into two sub-cavities,—an external and internal,—by an arch composed of thirty two pieces (the teeth) and their supports, which are covered by mucous membrane. All the walls of the entire cavity are covered by mucous membrane, imbedded in the substance of which are innumerable glands secreting mucus, which secretion is mixed with the saliva.

This is an important fact in the unfolding of the present view of the subject,—that there are contained in all of the mucosa of the mouth glands at all points secreting mucin.

The floor of the inner general cavity, inclosed by the teeth and gums, contains another,—a box-like cavity,—of which the gum-covered alveolar ridge and teeth form the greater part of the periphery.

The reflection of the mucous membrane of the base of the tongue, with that of the floor of the mouth, is its posterior boundary, the floor of the mouth, its movable base; and the under-surface of the tongue, its movable roof. Into this cavity are poured the secretions of the submaxillary and sublingual glands,—secretions containing comparatively large amounts of mucin and calcium phosphate and carbonate.

The great external cavity is a crescent having a thin body; its inner wall, the teeth and alveolar walls; its outer encasement, the soft tissues; the mucous membrane covering the orbicularis oris, buccinator, and the other lip-moving muscles.

The walls of these cavities have occasional periods for rest. When in movement the fluid contents are agitated, and the muscular boundaries by their action sweep the surfaces of the immovable walls.

The tip of the tongue has a lateral sweep from about the lingual surface of first molar of one side to that of first molar of the other.

The buccal and labial aspects of the anterior teeth are swept by the action of the extended and bent tip of the tongue. It will be seen that at the lingual aspect of the teeth, at the junction of gum and teeth, the contact of the tongue with gum and teeth leaves a V-shaped space. There are also similarly shaped spaces made by the peculiar contact of the teeth themselves.

The palatal surfaces of the superior teeth are swept quite thoroughly by the tongue,—their labial and buccal aspects almost equally as well,—except that on the buccal surfaces the action ends at about the junction of the first and second superior molars.

The movement of the sides of the tongue sweep clear the lingual walls of the inferior molars, except at the angle of junction between the gums and the teeth.

The movements of the lips and cheeks sweep the anterior walls of the teeth, except at the cervices of the inferior incisors, and the upper incisors, and of the superior molars. It is seen, therefore, that the angular spaces marking the junctions of gums and teeth are less subjected to this cleansing by muscular action than are the surfaces in general. The same mechanical forces would tend to drive any pasty material into the interstices between the teeth.

In addition to this purely mechanical cleansing, the fluid contents of the mouth are, through the same movements, kept in agitation, and are driven about into all open spaces, cleansing by irrigation.

When the muscles are at rest, there is a space between the mucous membrane covering the buccal muscles and the buccal faces of the superior molars. There is formed a pocket, the line of contact of the cheek with the first molar marking its forward extremity, that with the third molar its posterior, the deepest part of the pocket being opposite the superior second molar, into which the secretion of the parotid gland is poured

After all of the food-debris which is removable by the mechanical cleansing through the movements of the tongue, and by the irrigating action of the fluids of the mouth, has been swept away, there will be found more or less remnants clinging to surfaces and packed into interstices.

After eating, man, in common with other animals, sets in action a series of muscular movements with a view to remove food-fragments. Extraordinary tongue movements are made; the buccal and labial muscles are worked alternately, removing mechanically much debris, the saliva aiding in washing some of the material away; and, in addition, a suction is created which draws from between well-formed teeth much of the waste material. But, despite this, the cleansing is not complete.

The next step is an examination of the nature, the composition of this debris.

A fact which has no doubt impressed most of us is, that it is only man, and animals fed upon the food used by man, that are affected, at least in any marked degree, by dental caries and concretions upon the teeth. It is a natural inference, therefore, that the sources of these disorders are in a great measure due to food-habit.

Among the undomesticated, and among some domesticated animals, the food is taken in a raw state. In the herbivora the food is mainly carbohydrates, inclosed in great part in the tough fibrous structures of plants. When grain is eaten, but a small amount of debris remains. During the mastication of these substances the fibrous structures of the food serve as a means for mechanical cleansing. The starchy foods do not appear to have a tendency to mass. The starch is gradually set free from its envelope and ptyalized, leaving a mass of gluten, coherent and not pasty, and from which no debris remains.

In the carnivora, fleshy foods, when eaten in a natural state, are in such a condition that the muscular and fibrous tissues have no disposition to break or tear in fine fragments, and they thus have little or no tendency to pack, even were the teeth of carnivora not shaped as they are, to disfavor accumulations of debris.

The operation of cooking produces radical changes in the condition of foods. The cellulose covering of the cells, inclosing starch-granules, is burst; many varieties of starchy food are reduced to a pasty condition; the fibrous stems of plants are made friable,—a condition favoring retention in interstices. Human beings use a greater proportion of sugar than other animals; that is, use as a raw material a chemical substance which, with most animals, is a product of partial digestion.

Fleshy foods, by cooking, are made more tender and friable,—the solid fats are melted so that the neutral fats, palmitan and stearin, are taken in part as semi-liquids, not solids; and, it may be, have some alteration of their chemical composition. So that after mastication, small portions of muscular tissue and of hardened fats adhere to the surfaces of the teeth and pack into spaces.

It is doubtless the fact that saponaceous dentifrices, by removing these fatty deposits, give to the mouth a sense of cleanliness, which prompts many persons to use these preparations in preference to simple powders.

Dr. Miller's researches as to the etiology of dental caries, together with the fact that this is the most common of all diseases affecting mankind and, occasionally, lower animals, leave no doubt as to the primary cause of the disease. It is due to the action of a solvent, lactic acid; this body the product of a specific fermentation, set up through the agency of definite micro-organisms which, according to Pasteur, in a mixture containing nitrogenous material and carbohydrates, split the latter up into lactic acid: $C6H12 O6$ becoming $2 C3H6O3$; the nitrogenous matter serving for the nutrition of the organisms. Troussart also mentions specifically that lactic fermentation requires the presence of proteid matters. He also calls attention to the common bacteriological datum, that the fermentive action is self-limited; that it is checked by formation of lactic acid. Also, that a slightly alkaline medium is most suitable for the propagation of the lactic ferment.

Unquestionably in some mouths the lactic fermentation is accompanied or followed by more or less of butyric fermentation. Collections of food debris form the foci of of development for the fermentation inaugurated by these organisms; so that the use of food leaving debris which masses and packs in inaccessible places, is the primary factor, the predisposing cause, in the fermentation. The butyric fermentation follows upon the lactic, and in many cases, food deposits long retained are found to be the foci of putrefactive decomposition.

In connection with the matter, I have noted that in some gingival disorders the putrefactive decomposition appears to occur, without the transition fermentations.

Food which does not leave debris about and between the teeth offers no soil for the development of the micro-organisms. As the operation of cooking is that producing this difference of condition in food-stuffs, it is that which is responsible, at least in a great measure, for the production of lactic fermentations in the oral cavity.

Differences of oral condition no doubt modify the activity of fermentative processes, hence in the amount of acid formed. Disease-conditions, whether purely local or manifestations of other disorders,—any grade or variety of catarrhal stomatitis,—form a suitable soil for the activity of ferments.

In addition to this result, catarrhal processes are associated with a swelling of the tissues, an increased exudation into the perivascular spaces causes the epithelial cells to swell by imbibition; the secretion of the glands is increased and modified; there is a visible swelling of the membrane affected, about the necks of the teeth. According to the grade and variety of the stomatitis, the swelling causes the formation of pockets, of variable depth, due to what is known as the dental ligament resisting detachment and the mucous membrane swelling around it.

We have examined now two of the factors involved,—first, the fluid, the *origo materiæ*; second, the cavity into which this is poured, together with contributions made by the glands of its walls, and also some of the adventitious matters which are suspended in the combined fluids.

The condition is: A fluid containing, as its greater portion of solids, calcium salts and mucin, in solution, is poured into the cavity, containing fluid; at several situations of the cavity, lactic fermentation is in progress. In addition to this, the places of lactic fermentation have, as one wall, a membrane containing mucin-secreting glands, and the general fluid is in consequence made acid in reaction.

This is a condition, some degree of which is almost universal. It is subject to gradations in different individuals, and, indeed, in the same individual.

The third inquiry as to the nature of the products discussed. These are accretions varying in degree of hardness from that of a granular mass, less hard than plaster of Paris, to the stony concretions found at times beneath the borders of the gum line, and approximating white coral in hardness. In color, they range from light yellow to dark green. In amount, from small scaly particles to masses in which several teeth are imbedded, so as to make the outlines of the several teeth indistinguishable. The lighter the collections are in color, the softer or more friable they are; the darker they are, the more dense; or the more dense they become, the harder they are.

There appear to be three classes of the calculi,—those of the variety usually found upon the inferior incisors; second, those upon the buccal faces of molars; and third, the green deposits, some part or all of which are beneath the margin of the gum. Judging from my observations thus far, I believe the latter are due, at least in great part, to the altered secretion of the gingival glands; some alteration in the usual composition of the colloid medium producing modification of the calculus. They are formed more slowly than the others, and it appears that they are produced in some cases *after* the formation of yellow calculi. My own interpretation of this is, that the yellow calculus causes nutritive disturbance in the small glands, and according to the grade of this disturbance, ranging from irritative activity to pus-formation, will be the character of the resulting calculus.

The light colored and softer masses have a predilection for two positions at the linguo-cervical aspects of the inferior incisors, to some extent the labio-cervices of the same teeth, on a line with the openings of the ducts of the submaxillary and sublingual glands.

Upon the buccal surfaces of the superior molars, opposite the opening of the ducts of the parotid glands, the dark, hard deposits are usually, or almost always, confined to the crevices of the teeth projecting into or from the sharp angle of junction between the teeth and gums.

It is not known to the writer that any separate analyses have been made of these several varieties of calculi. Dark-colored, dense, and hard specimens tested by the writer have given, upon heating, a strong animal odor, leaving a debris of some mineral matter and carbon; the mineral matter perfectly soluble in dilute nitric acid, with but little effervescence, calcium phosphate probably. Occasionally there was a faint response to the murexid test. The yellow deposits behaved in a similar manner. The crude analysis of the writer showed no marked difference in the composition of the several varieties.

Harlan, "American System of Dentistry," vol. ii, quoting Schehevetskey, gives as an analysis of the deposits:

| | |
|---|---|
| Water and organic matter . | 22.07 |
| Magnesium phosphate . . | 1.07 |
| Calcium phosphate . . | 67.18 |
| " carbonate . . | 8.13 |
| " fluoride . . | 1.55 |

Nine-tenths of the deposits is composed of calcium phosphate, water, and organic matter. It has been determined that the 22.07 per cent. is composed in great part of mucin. (I have seen this stated, but cannot recall now whose analysis mentions the mucin.)

The problem is to determine the interactions in the two factors which stand in causal relation to the third. Why should these deposits occur? The following experiment appears to furnish a reasonable basis for the formation of a hypothesis which, it is hoped, will through further investigation ultimately throw more light on this subject.

Place in a test-tube two inches or more of saliva,— a fluid holding in suspension lime-salts, mucin and an amount of $CO_2$. Over this pour one or two drops of a ten per cent solution of lactic acid. Almost immediately a turbidity of the contents of the test-tube is noticed. This gradually but quite rapidly resolves itself into a coagulum, extending as a core through the fluid; it slowly contracts, and, in contracting, rises until it floats upon the surface. If the saliva has been ejected for some time, the salts of calcium are beginning to precipitate. In the condensation of the coagulum the suspended material is entangled in its meshes, and rises as part of the coagulum; perhaps a portion of the calcium phosphate being converted into a lacto-phosphate.

(To be continued.)

